# Dataset on Islamic school teachers’ organizational commitment as factors affecting job satisfaction and job performance

**DOI:** 10.1016/j.dib.2021.107181

**Published:** 2021-05-29

**Authors:** Deny Setiawan Wayoi, Margana Margana, Lantip Diat Prasojo, Akhmad Habibi

**Affiliations:** a Student of Doctorate Program of Educational Management, Universitas Negeri Yogyakarta, Indonesia; b Universitas Negeri Yogyakarta, Indonesia; c Universitas Jambi, Indonesia

**Keywords:** Organizational commitment, Job performance, Job satisfaction, Islamic education, Survey

## Abstract

This dataset aims to examine the effect of Organizational Commitment (OC) factors on Job Performance (JP) and Job Satisfaction (JS) at Islamic schools in Indonesia. The data propose that OC factors (Affective Commitment (AC), Normative Commitment (NC), and Continuance Commitment (CC)) have significant influences on JS and JP. Through the survey approach, 387 responses were obtained from Islamic schoolteachers in Indonesia. Face and content validity were initiated after the adaptation of the survey instrument. Further, a Partial Least Squares Structural Equation Model (PLS-SEM) was used to test the reliability and validity of the measurement model. For this purpose, the statistical process presents reflective indicator loadings, internal consistency reliability, and convergent and discriminant validity. Future researchers may reuse this dataset as a potential instrument to measure factors affecting job satisfaction and job performance within the context of education.

## Specifications Table

**Table:** 

Subject	Education
Specific subject area	Islamic education, OC, JS, and JP
Type of data	Table Figure
How data were acquired	Face and content validity, survey, and PLS-SEM
Data format	Raw Analyzed Filtered
Parameters for data collection	The instrument includes demographic information, OC (AC, NC, and CC), JS, and JP
Description of data collection	The instrument was adapted, back-translated, and validated. The analysis was conducted through PLS-SEM procedure for the measurement model
Data source location	Province: Yogyakarta, Jambi Country: Indonesia Latitude and longitude (and GPS coordinates) for collected samples/data: .7893° S, 113.9213° E
Data accessibility	On a public repository Repository name: Mendeley Data Data identification number: 10.17632/dzy22g86tt.2 Direct URL to the data: https://data.mendeley.com/datasets/dzy22g86tt/2

## Value of the Data

• The dataset presents a validation process of a survey of OC factors affecting JS and JP in the context of Islamic educational institutions.
• The data are useful for educational regulators in facilitating proper policies regarding OC to improve their teachers JS and JP.
• The accessible dataset could contribute to future researchers interested in doing research on similar topics

## 1 Data Description

**Fig. 1:**
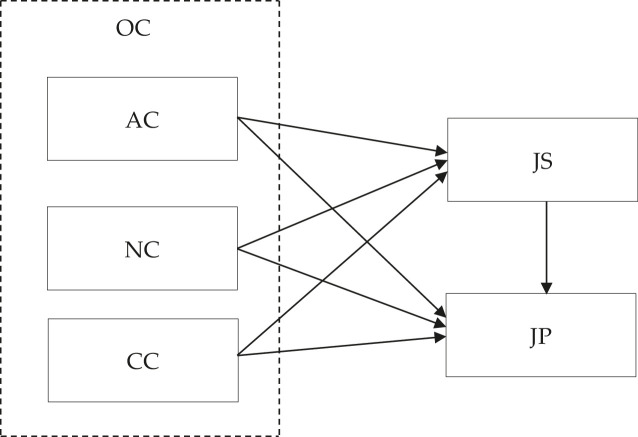
Proposed model.

**Fig. 2:**
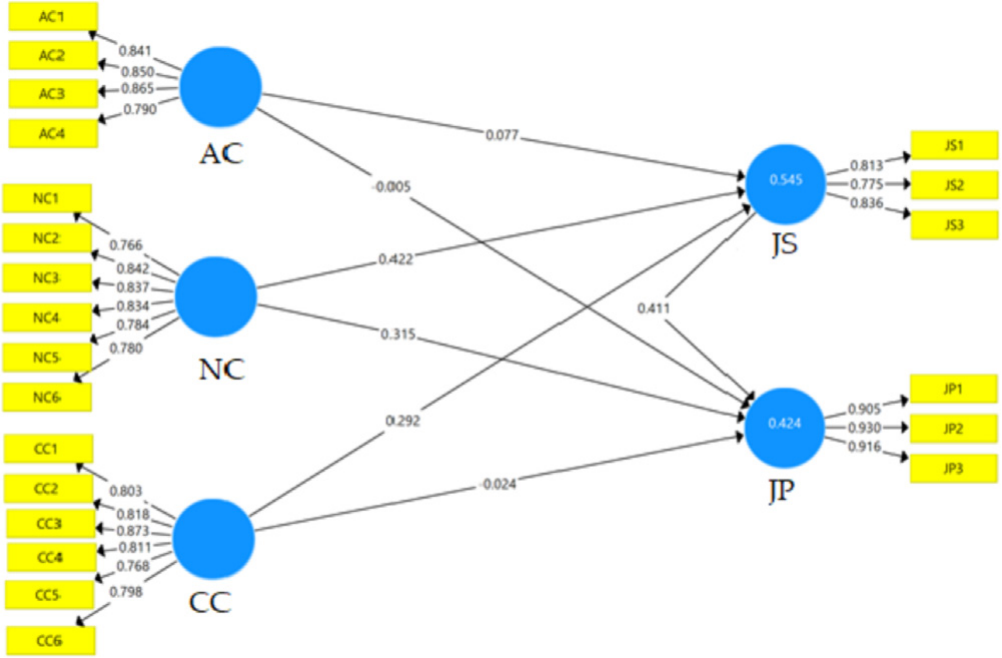
Measurement model.

This dataset proposes that Organizational Commitment (OC) factors that consist of Affective Commitment (AC), Normative Commitment (NC), and Continuance Commitment (CC) have significant influences on Job Performance, (JP), Job Satisfaction (JS). OC is defined as a degree to which teachers associates with their organization, the relative strength of their school involvement. AC is described as teachers’ inner attachment to the school they teach, CC refers to the perception of costs, related to leaving the organization, and NC stands for the sense of responsibility possessed by the participants for the schools where they work. JP represents a term that refers to the quality of work of teachers in their profession. Meanwhile, JS is a pleasurable emotional state that results from the teaching experience that the participants have. The dataset includes two sections; demographic information and main survey. The demographic questions consist of location, gender, teaching experience, and school level (Table 1). The main survey has three exogenous and two endogenous constructs (Fig. 1). Three exogenous constructs included in OC measured from 1 = very disagree to 5 = very agree are AC (6 items), NC (6 items), and CC (6 items), adapted from previous academic research [1,2]. Meanwhile, two endogenous constructs are JS (3 items, 5 = mostly true; 1 = mostly false) and JP (3 items, 1 = very poor; 5 = very good) [2,3]. Table 2 informs the Mean, Standard Deviation, Skewness and Kurtosis of the data. Table 3 provides the information of the three assessments of measurement model (reflective indicator loadings, internal consistency reliability, and convergent validity). Tables 4 and 5 show the discriminant validity through the assessment of Fornell-Larcker criterion and cross-loading. Fig. 2 exhibits the measurement model of the dataset. The raw dataset and instrument are accessible on https://data.mendeley.com/datasets/dzy22g86tt/2.

**Table 1:** Demographic information (n.387).

Demographic	n	%
*Location*		
Yogyakarta	264	68.22
Jambi	123	31.78
*Gender*		
Female	226	58.40
Male	161	41.60
*Teaching experience*		
<5 years	96	24.81
5-10 years	86	22.22
>10 years	205	52.97
*School level*		
Senior high	262	67.70
Junior high	125	32.30

**Table 2:** Mean, SD, Skewness, and Kurtosis.

			Skewness		Kurtosis	
	M	SE		Std. Error		Std. Error
AC1	4.3669	.67888	-.957	.124	1.386	.247
AC2	4.5297	.62836	-1.309	.124	2.415	.247
AC3	4.4212	.70223	-1.252	.124	2.307	.247
AC4	4.3127	.70741	-.927	.124	1.217	.247
AC5	4.0491	.82397	-.762	.124	.832	.247
AC6	3.8010	1.05996	-.829	.124	.269	.247
CC1	4.3928	.67198	-.814	.124	.183	.247
CC2	4.2713	.75239	-.639	.124	-.479	.247
CC3	4.3979	.68424	-.846	.124	.126	.247
CC4	4.3979	.73885	-1.097	.124	.973	.247
CC5	3.9819	.82837	-.324	.124	-.702	.247
CC6	4.1628	.73538	-.423	.124	-.544	.247
NC1	4.0155	.74442	-.176	.124	-.766	.247
NC2	4.2041	.73208	-.617	.124	.224	.247
NC3	4.2196	.69478	-.418	.124	-.522	.247
NC4	4.2171	.65532	-.425	.124	.003	.247
NC5	4.4922	.59973	-.805	.124	.056	.248
NC6	4.0413	.80693	-.492	.124	-.182	.247
JS1	4.3618	.68544	-.705	.124	-.245	.247
JS2	3.7700	1.07314	-.657	.124	-.309	.247
JS3	3.9819	.91174	-.686	.124	.015	.247
JP1	3.9974	.79343	-.527	.124	.097	.247
JP2	3.8140	.88833	-.362	.124	-.380	.247
JP3	3.8088	.86355	-.446	.124	.090	.247

**Table 3:** Reflective indicator loadings, internal consistency reliability, and convergent validity.

		Load	α	CR	(AVE)
AC	AC1	.841	.857	.903	.700
	AC2	.850			
	AC3	.865			
	AC4	.790			
CC	CC1	.803	.896	.921	.660
	CC2	.818			
	CC3	.873			
	CC4	.811			
	CC5	.768			
	CC6	.798			
JP	JP1	.905	.906	.925	.841
	JP2	.930			
	JP3	.916			
JS	JS1	.813	.736	.850	.653
	JS2	.775			
	JS3	.836			
NC	NC1	.766	.893	.918	.652
	NC2	.842			
	NC3	.837			
	NC4	.834			
	NC5	.784			
	NC6	.780

**Table 4:** Fornell-Larcker criterion.

	AC	CC	JP	JS	NC
AC	.837				
CC	.605	.812			
JP	.400	.522	.917		
JS	.526	.693	.617	.808	
NC	.646	.841	.586	.717	.808

**Table 5:** Cross-loading.

	AC	CC	JP	JS	NC
AC1	**.841**	.486	.319	.417	.538
AC2	**.850**	.541	.340	.465	.527
AC3	**.865**	.558	.347	.473	.565
AC4	**.790**	.433	.333	.400	.532
CC1	.635	**.803**	.428	.582	.665
CC2	.498	**.818**	.433	.585	.672
CC3	.559	**.873**	.410	.586	.704
CC4	.446	**.811**	.414	.559	.595
CC5	.365	**.768**	.427	.509	.682
CC6	.434	**.798**	.433	.551	.779
JP1	.388	.544	**.905**	.589	.571
JP2	.339	.429	**.930**	.544	.489
JP3	.370	.457	**.916**	.562	.546
JS1	.566	.608	.477	**.813**	.665
JS2	.320	.471	.444	**.775**	.495
JS3	.371	.589	.569	**.836**	.566
NC1	.422	.669	.474	.543	**.766**
NC2	.472	.725	.471	.621	**.842**
NC3	.474	.730	.454	.568	**.837**
NC4	.606	.675	.494	.601	**.834**
NC5	.624	.669	.422	.586	**.784**
NC6	.529	.605	.520	.551	**.780**

## 2 Experimental Design, Materials and Methods

For the scale development, a 2-phase procedure was implemented. In phase 1, the adaptation and translation were conducted. The adaptation of previous sources of literature was made [1], [2], [3] followed by the translation of the scale. The scale was translated using a back-translation method from English to Indonesian and Indonesia to English that involved 2 translation experts. In phase 2, face and content validity were conducted with two sessions of discussions with 5 users and 5 experts to evaluate the scale for the appropriateness of context and setting. We collected the data from September to December 2020 through a simple random sampling. We randomly selected a subset of participants from the population who are Islamic school teachers in two Indonesian provinces, Yogjakarta and Jambi. Every teacher has an equal opportunity of being opted as the participant. After converting the data in Microsoft Excel, the normality assessment was done by calculating Skewness and Kurtosis in SPSS 23, in which the values should be between -2 to + 2 [4]. All Skewness and Kurtosis values are in the range of the threshold; Skewness (AC4, -1.039 to NC1, -176) and Kurtosis (AC4, 2.415 to NC1,-766) (Table 1). Further, the analysis of the data was conducted through the measurement model; four assessments (reflective indicator loadings, internal consistency reliability, convergent, and discriminant validity) were reported within the approach of PLS-SEM in SmartPLS 3.2. The reflective indicator loading should be .708 or higher. Table 2 performs all loading values that fulfill the threshold (.775-.930). Cronbach's alpha and Composite Reliability (CR) of greater than .700 should be applied for the internal consistency [5,6]. The Cronbach's alpha values of this dataset range from .736 to .906; similarly, the CR values are between .850 and .925 [7,8]. Convergent validity was reported through Average Variance Extracted (AVE); the values are recommended to be .500 or higher [8]. The AVE values range from .652 to .841 (Table 2). By using the Fornell-Larcker and cross-loading, the discriminant validity was evaluated. The AVE values of a construct should be less than the shared variance for other constructs for the Fornell-Larcker. The values of every construct are less than it's shared variance (Table 4). When loading on a construct is greater than those of other constructs; cross-loading values, the discriminant validity is reported. All indicators’ values (bold) of every construct were above the values of all their cross-loadings (Table 5). Two items were dropped due to low loading values (AC5 and AC6). The model consists of five constructs with 22 indicators (Fig. 2).

## Ethics Statement

Informed consent was obtained for the data collection and the participation was voluntary. The survey was anonymous that did not include any personal information of the participants.

## CRediT Author Statement

**Deny Setiawan Wayoi:** Conceptualization, Methodology, Software, Data curation, Investigation; **Margana Margana**: Conceptualization, Supervision; **Lantip Diat Prasojo:** Conceptualization, Supervision; **Akhmad Habibi**: Software, Validation, Visualization, Writing original draft preparation.

## Declaration of Competing Interest

The authors declare that they have no known competing financial interests or personal relationships which have or could be perceived to have influenced the work reported in this article.

## References

[1] Meyer J.P., Alien N.J. (1991). A three-component conceptualization of organizational commitment. Hum. Resour. Manag. Rev..

[2] Fu W., Deshpande S.P. (2014). The impact of caring climate, job satisfaction, and organizational commitment on job performance of employees in a china’s insurance company. J. Bus. Ethics.

[3] Celluci (1978). Measuring Managerial Satisfaction: A Manual for the MJSQ.

[4] Habibi F.D.Yusop, Razak R.A. (2020). The dataset for validation of factors affecting pre-service teachers’ use of ICT during teaching practices: Indonesian context. Data Br..

[5] Habibi F.D.Yusop, Razak R.A. (2020). The role of TPACK in affecting pre-service language teachers’ ICT integration during teaching practices: Indonesian context. Educ. Inf. Technol..

[6] Hair J.F., Sarstedt M., Ringle C.M. (2019). Rethinking some of the rethinking of partial least squares. Eur. J. Mark..

[7] Prasojo L.D., Habibi A., Yaakob M.F.M., Pratama R., Yusof M.R., Mukminin A., Suyanto F.HanumH. (2020). Dataset relating to the relationship between teacher self-concept and teacher efficacy as the predictors of burnout: a survey in Indonesian education. Data Br..

[8] Hair J.F., Risher J.J., Sarstedt M., Ringle C.M. (2019). When to use and how to report the results of PLS SEM. Eur. Bus. Rev..

